# Field evaluation of a *Pf*HRP-2/pLDH rapid diagnostic test and light microscopy for diagnosis and screening of falciparum malaria during the peak seasonal transmission in an endemic area in Yemen

**DOI:** 10.1186/s12936-016-1103-2

**Published:** 2016-01-28

**Authors:** Lina M. Q. Alareqi, Mohammed A. K. Mahdy, Yee-Ling Lau, Mun-Yik Fong, Rashad Abdul-Ghani, Arwa A. Ali, Fei-Wen Cheong, Rehab Tawfek, Rohela Mahmud

**Affiliations:** Department of Parasitology, Faculty of Medicine, University of Malaya, 50603 Kuala Lumpur, Malaysia; Tropical Disease Research Center, University of Science and Technology, Sana’a, Yemen; Department of Parasitology, Faculty of Medicine and Health Sciences, Sana’a University, Sana’a, Yemen; Yemeni-Swedish Hospital, Ministry of Public Health and Population, Taiz, Yemen

**Keywords:** *Plasmodiumfalciparum*, Microscopy, Rapid diagnostic test, Nested PCR, Yemen

## Abstract

**Background:**

Malaria is a public health threat in Yemen, with 149,451 cases being reported in 2013. Of these, *Plasmodium falciparum* represents 99 %. Prompt diagnosis by light microscopy (LM) and rapid diagnostic tests (RTDs) is a key element in the national strategy of malaria control. The heterogeneous epidemiology of malaria in the country necessitates the field evaluation of the current diagnostic strategies, especially RDTs. Thus, the present study aimed to evaluate LM and an RDT, combining both *P. falciparum* histidine-rich protein-2 (*Pf*HRP-2) and *Plasmodium* lactate dehydrogenase (pLDH), for falciparum malaria diagnosis and survey in a malaria-endemic area during the transmission season against nested polymerase chain reaction (PCR) as the reference method.

**Methods:**

A household-based, cross-sectional malaria survey was conducted in Mawza District, a malaria-endemic area in Taiz governorate. A total of 488 participants were screened using LM and *Pf*HRP-2/pLDH RDT. Positive samples (160) and randomly selected negative samples (52) by both RDT and LM were further analysed using 18S rRNA-based nested PCR.

**Results:**

The sensitivity, specificity, positive predictive value (PPV), and negative predictive value (NPV) of the RDT were 96.0 % (95 % confidence interval (CI): 90.9–98.3), 56.0 % (95 % CI: 44.7–66.8), 76.3 % (95 % CI: 69.0–82.3), and 90.4 % (95 % CI: 78.8–96.8), respectively. On the other hand, LM showed sensitivity of 37.6 % (95 % CI: 29.6–46.3), specificity of 97.6 % (95 % CI: 91.7–99.7), PPV of 95.9 % (95 % CI: 86.3–98.9), and NPV of 51.3 % (95 % CI: 43.2–59.2). The sensitivity of LM dropped to 8.5 % for detecting asymptomatic malaria. Malaria prevalence was 32.8 % (32.1 and 37.5 % for ≥10 and <10 years, respectively) with the RDT compared with 10.7 % (10.8 and 9.4 % for age groups of ≥10 and <10 years, respectively) with LM. Among asymptomatic malaria individuals, LM and RDT-based prevalence rates were 1.6 and 25.6 %, respectively. However, rates of 88.2 and 94.1 % of infection with *P. falciparum* were found among patients who reported fever in the 48 h prior to the survey by LM and *Pf*HRP-2/pLDH RDT, respectively.

**Conclusions:**

The *Pf*HRP-2/pLDH RDT shows high sensitivity for the survey of falciparum malaria even for asymptomatic malaria cases. Although the RDT had high sensitivity, its high false-positivity rate limits its utility as a single diagnostic tool for clinical diagnosis of malaria. On the other hand, low sensitivity of LM indicates that a high proportion of malaria cases is missed, underestimating the true prevalence of malaria in the community. Higher NPV of *Pf*HRP-2/pLDH RDT than LM can give a straightforward exclusion of malaria among febrile patients, helping to avoid unnecessary presumptive treatments.

## Background

Malaria is a public health threat causing morbidity and mortality in Yemen, with *Plasmodium falciparum* being the predominant species responsible for almost 99 % of cases. It is estimated that 43 % of the population are at high risk and a total of 63,484 microscopy-confirmed and 39,294 rapid diagnostic test (RDT)-confirmed cases were reported in 2013 [1]. Yemen is in the control phase, and the adopted malaria control strategies include distributing insecticide-treated nets, indoor residual spraying, prompt diagnosis and treatment with artemisinin-based combination therapy [2].

Light microscopy (LM) is still the cornerstone of malaria diagnosis in Yemen, especially in hospitals. However, LM has low sensitivity for detection of low parasite densities, is time-consuming and requires skilled technicians and good reagents [2, 3]. Therefore, it may not reflect the submicroscopic infectious reservoir in Yemen, which is still neglected and needs to be estimated if malaria elimination in the country is to be achieved [4]. RDTs have been introduced as an alternative to LM, especially when good LM practice cannot be maintained or is not available. RDTs that target *P. falciparum* histidine-rich protein-2 (*Pf*HRP-2) have the highest and most consistent detection rate [5]. In contrast, *Plasmodium* lactate dehydrogenase (pLDH) detects all *Plasmodium* species and is usually combined with *Pf*HRP-2 for malaria screening in areas endemic with multiple species [6, 7]. The National Malaria Control Programme (NMCP) has been using RDTs for malaria diagnosis and field surveys since 2007 [2, 8]. Although the World Health Organization (WHO) has provided comparative data on the performance of RDTs that can be used for procurement decision, it is well recognized that clinical sensitivity of RDTs depends on the epidemiology of malaria in the target population [5], which imposes field evaluation of such tests. In Yemen, malaria is unstable, seasonal and affected by topography and rainfall. The country has been stratified with respect to malaria endemicity into four strata that are different in altitude, intensity, length, and season of transmission and even in the predominant vector species [2]. This heterogeneous epidemiology of malaria may affect the performance of RDTs, necessitating the need for their evaluation in the four strata. In Yemen, only two previous studies evaluated the performance of *Pf*HRP-2-based RDTs against LM as the ‘gold standard’ [9, 10]. It is, however, noteworthy that false-negativity of LM limits its accuracy as reference method. Polymerase chain reaction (PCR) is more sensitive than LM and RDTs for detecting malaria in epidemiological studies assessing asymptomatic carriers in low endemicity settings [11–13]. This is the first community-based survey to evaluate the performance of LM and a *Pf*HRP-2/pLDH RDT for malaria diagnosis against PCR as the reference method during the transmission season in a malaria-endemic area in Taiz governorate.

## Methods

### Study design and area

**Fig. 1:**
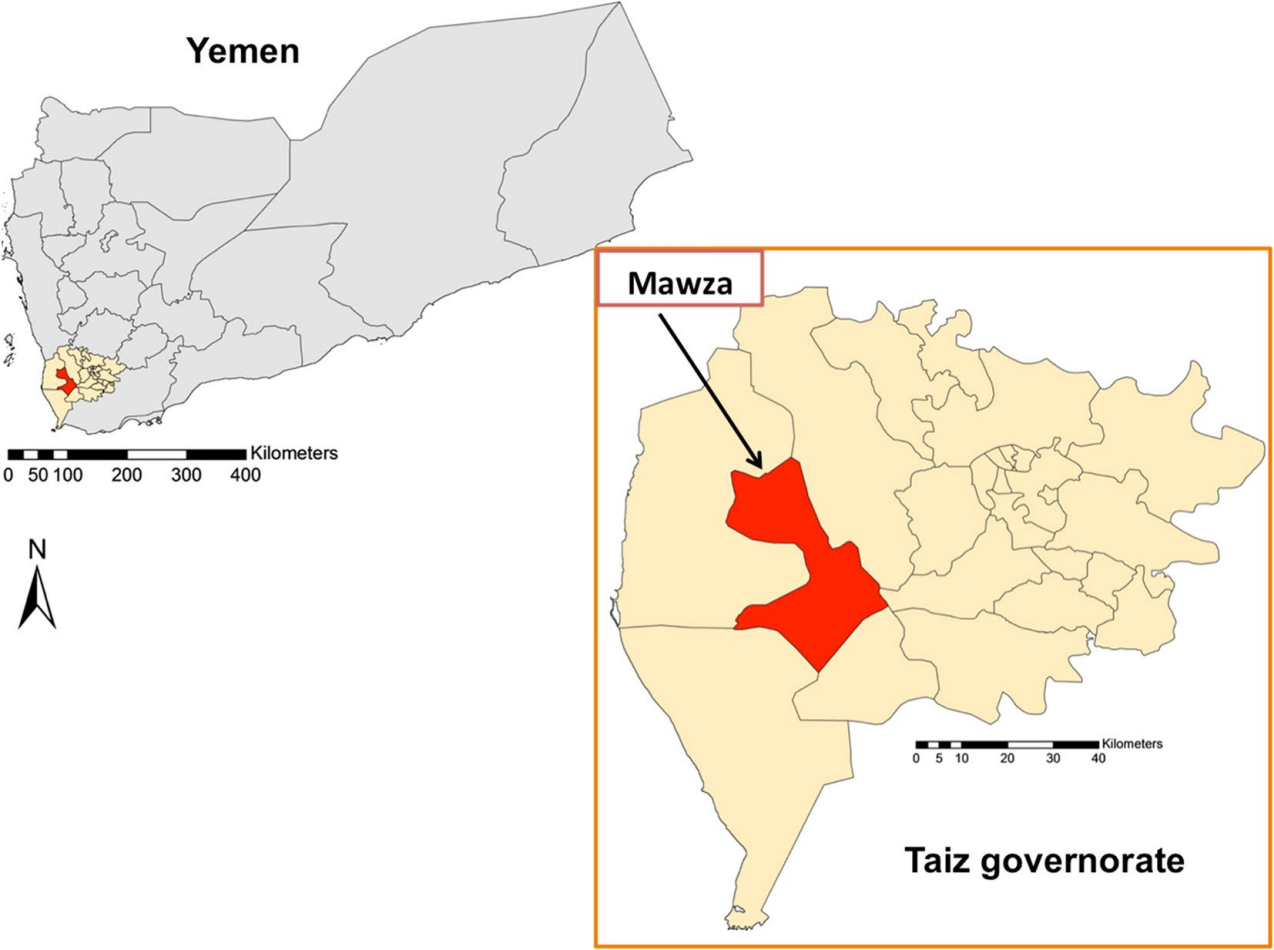
Map of Yemen showing the study area

The present study is a cross-sectional study in Mawza District, which is a malaria-endemic area located in Taiz governorate, south of Yemen (Fig. 1). Its total area is about 665 sq km and is inhabited by a total population of 119,818 people. It has a coastal climate that is warm in winter and hot in summer, with irregular heavy torrents of rainfall. The mean temperature is 29 °C, and the humidity reaches 67 %. Peak malaria transmission occurs between October and April. The area is classified by the NMCP as belonging to stratum one, which has an altitude of less than 600 m and is characterized by high malaria transmission [2, 8, 14].

### Ethical clearance and field survey

The protocol of the study was approved by the Ethical Committee of the University of Science and Technology, Yemen. This study recruited 488 participants through a household-based survey in the period from October 2013 to April 2014. All family members, of all age groups and both sexes, were invited to participate on a voluntary basis from randomly selected houses in the study area. Signed or thumb-printed informed consent was taken from the participants or their guardians before sample collection and after a clear explanation of the study objectives. Data on the age of participants, history of anti-malarial drug intake 1 week prior to the study, and history of fever 48 h before the study were collected using a pre-designed questionnaire. Whole blood samples (about 5 ml) were collected in EDTA tubes from all participants that were tested for malaria by using both RDT and LM. Haemoglobin was measured using Sysmex KX-21N Hematology Analyzer (Sysmex Corp, Chuo-Ku, Kobe, Japan). RDT-positive participants were treated according to the national malaria treatment policy of the Ministry of Health and Population, Yemen. For molecular investigation, blood spots were blotted onto Whatmann^®^ 3MM filter papers, air-dried and kept in separate plastic bags at room temperature until DNA extraction.

### LM and RDT screening

Thick and thin blood films (one slide per sample) were prepared and stained with Giemsa for approximately 20 min according to standard procedures. Thick films were examined using a light microscope by a qualified laboratory technician for a minimum of 100 high-magnification fields before being recorded as negative for malaria parasites. These films were then blindly examined by an independent malaria microscopist for the confirmation of the first examination. A third blinded examination was conducted for those films having discordant results between the first and second examiners. The results for the third examination were regarded as final. All microscopists in this study were trained by the NMCP. Parasite density per μL of blood was estimated for each sample by counting the number of parasites against 200 white blood cells (WBC), assuming a standard mean WBC count of 8000/μL blood [15]. Samples were then categorized into three groups based on parasite density; low (1–999 parasites/µL), moderate (1000–9999 parasites/µL) and high (>10,000 parasites/μL) [16].

The SD Bioline^®^ Malaria Antigen *Pf/*Pan test, product code: SD05FK63 (Standard Diagnostics, Inc, Kyonggi, Korea) that combines the detection of *Pf*HRP-2 and pLDH was used based on the WHO recommendations as being one of the ten top-performing RDTs [17]. Test kits were kept at room temperature, in compliance with the manufacturer’s instructions, and tests were performed following the manufacturer’s instruction by using 5 µL of whole blood samples. RDT results were read and interpreted within 15–30 min according to the manufacturer’s instructions.

### DNA extraction and molecular detection

Genomic DNA was extracted from dried blood spots by using DNeasy^®^ Blood and Tissue Kit (QIAGEN, Hilden, Germany) according to the manufacturer’s instructions and kept at −20 °C until used. Genus- and species-specific nested PCR assays based on the 18S rRNA gene were used to detect and identify *P*. *falciparum* [18]. PCR for primary and secondary reactions were run in a total of 25 μL reaction mixture containing 4 μL template, 200 μM of each deoxynucleotide triphosphate (dNTP), 4 mM MgCl_2_, 200 nM of each primer and 1 U of *Taq* polymerase. The cycling conditions for primary PCR were as follows: an initial denaturation step at 94 °C for 4 min, then 35 cycles at 94 °C for 1 min, annealing at 55 °C for 1 min and extension at 72 °C for 1 min, and a final extension at 72 °C for 10 min. Secondary PCR used similar cycling conditions except that the annealing temperature was increased to 58 °C. PCR products were separated by electrophoresis on 1.5 % agarose gel and stained with SYBR^®^ Safe DNA Gel Stain (Invitrogen™, CA, USA).

### Statistical analysis

Data obtained were entered and analysed using the Statistical Package for Social Sciences (SPSS) version 20.0 (SPSS Inc, Chicago, IL, USA). The sensitivity, specificity, positive predictive value (PPV), and negative predictive value (NPV) of the RDT and LM were calculated against the nested PCR with their 95 % confidence intervals (CIs). Cohen’s kappa coefficient (*Kc*) was used to assess agreement between results obtained by two different tests [19], and the strength of agreement was scaled as follows: slight (*Kc* = 0.01–0.20), fair (*Kc* = 0.21–0.40), moderate (*Kc* = 0.41–0.60), substantial (*Kc* = 0.61–0.8), or almost perfect (*Kc* = 0.81–1) [20]. Statistical significance was considered at p < 0.05.

## Results

### Characteristics of study subjects and prevalence of of malaria based on LM and PfHRP-2/pLDH RDT

Of 488 participants, 35.2 % were males and 64.8 % were females. The median age of participants was 26 years old (interquartile range: 22–28 years). About 10.5 % (51/488) of participants had self-reported fever. The majority of participants (42 %; 205/488) were anaemic, having haemoglobin levels less than 11 g/dL. LM detected falciparum malaria among 10.7 % (10.8 and 9.4 % for age groups of ≥10 and <10 years, respectively) of patients. Of them, 9.6 % (5/52) had a low parasitaemia, 32.7 % (17/52) had a moderate parasitaemia, 42.3 % (22/52) had a high parasitaemia and 15.4 % (8/52) showed only gametocytes. On the other hand, the overall prevalence of *P. falciparum* among the inhabitants of Mawza District was 32.8 % based on *Pf*HRP-2/pLDH RDT (32.1 and 37.5 % for ≥10 and <10 years, respectively). The prevalence was four times higher among children <10 years old with the RDT than with LM. Asymptomatic falciparum malaria among the study population was found to be 1.6 % with LM compared with 25.6 % with the RDT. However, rates of 88.2 and 94.1 % of infection with *P. falciparum* were found among patients who reported fever in the 48 h prior to the survey by LM and PfHRP-2/pLDH RDT, respectively (Table 1).

**Table 1:** Prevalence of *Plasmodium falciparum* based on LM and *Pf*HRP-2/pLDH RDT screening in Mawza District, Taiz Governorate, Yemen

		LM		*Pf*HRP-2/pLDH RDT	
	No	n	% (95 % CI)	n	% (95 % CI)
Overall	488	52	10.7 (8.2–13.7)	160	32.8 (28.8–37.1)
Age (years)					
≥10	424	46	10.8 (8.2–14.2)	136	32.1 (27.8–36.7)
<10	64	6	9.4 (4.4–19.0)	24	37.5 (26.7–49.8)
Fever 48 h prior to screening					
Yes	51	45	88.2 (76.6–94.5)	48	94.1 (84.1–98.0)
No	437	7	1.6 (1.0–3.3)	112	25.6 (21.8–29.9)
History of anti-malarial drug intake 1 week prior to the survey					
Yes	157	20	13.0 (9.0–19.0)	76	48.0 (41.0–56.0)
No	331	32	10.0 (7.0–13.0)	84	25.0 (21.0–30.0)

*n* number positive, *CI* confidence interval *LM* light microscopy *PfHRP-2* *P. falciparum* histidine-rich protein-2, *pLDH* *Plasmodium* lactate dehydrogenase, *RDT* rapid diagnostic test

### Comparison between *Pf*HRP-2/pLDH RDT and LM

Table 2 shows that *Pf*HRP-2/pLDH RDT detected all LM-positive cases of falciparum malaria, irrespective of parasite density. Of the LM-negative samples, 24.6 % (107/435) were RDT-positive. RDT and LM showed a fair agreement (77.8 %; *Kc* = 0.379, p < 0.001) for the detection of *P. falciparum* among all participants. However, the two tests had a substantial agreement (94.1 %; *Kc* = 0.638, p < 0.001) for detecting the infection among febrile patients (Table 2).

**Table 2:** Comparison between *Pf*HRP-2/pLDH RDT and LM for detecting *Plasmodium falciparum* malaria in Mawza District, Taiz Governorate, Yemen

		LM				
	RDT	Positive	Negative	Total	% Agreement (*Kc*)	p value
Overall	Positive	52	108	160	77.9 (0.390)	<0.001
	Negative	0	328	328		
	Total	52	436	488		
Febrile	Positive	45	3	48	94.0 (0.638)	<0.001
	Negative	0	3	3		
	Total	45	6	51		
Afebrile	Positive	7	105	112	76.0 (0.090)	<0.001
	Negative	0	325	325		
	Total	7	430	437		

*PfHRP-2* *P. falciparum* histidine-rich protein-2, *pLDH* *Plasmodium* lactate dehydrogenase, *RDT* rapid diagnostic test, *LM* light microscopy0, *Kc* Cohen’s kappa coefficient;  % agreement was calculated by summation of the number of positives and negatives by both RDT and LM divided by the total number of cases

### Sensitivity, specificity, positive, and negative predictive values of *Pf*HRP-2/pLDH RDT against nested PCR

Compared with nested PCR, the *Pf*HRP-2/pLDH RDT had sensitivity of 96.0 % (95 % CI: 90.9–98.3) and specificity of 56.0 % (95 % CI: 44.7–66.8), PPV of 76.3 % (95 % CI: 69.0–82.3), and NPV of 90.4 % (95 % CI: 78.8–96.8). The two types of tests showed a moderate degree of agreement (79.8 %; *Kc* = 0.553, p < 0.001). In addition, the RDT maintained its high sensitivity for the detection of *P. falciparum* among children <10 years old, asymptomatic participants and those with history of anti-malarial drug intake. However, it showed low specificity, which dropped to about 30 % among people with history of anti-malarial drug intake (Table 3).

**Table 3:** Sensitivity, specificity, PPV and NPV of *Pf*HRP-2/pLDH RDT for detecting *Plasmodium falciparum* against nested PCR as a reference method

	PCR + ve	PCR + ve	PCR−ve	PCR−ve	Sensitivity	Specificity	PPV	NPV	% Agreement*
	RDT + ve	RDT−ve	RDT−ve	RDT + ve	% (95 % CI)	% (95 % CI)	% (95 % CI)	% (95 % CI)	(*Kc*)
Overall	119	5	47	37	96.0 (90.9–98.3)	56.0 (44.7–66.8)	76.3 (69.0–82.3)	90.4 (78.8–96.8)	79.8 (0.553)
Age (years) (n = 208)									
≥10	100	5	41	32	95.2 (89.3–97.9)	56.2 (44.1–67.8)	75.8 (67.8–82.3)	89.1 (76.4–96.4)	79.2 (0.545)
<10	19	0	6	5	100 (82.4–100)	54.6 (23.4–83.3)	79.2 (57.9–92.9)	100 (54.1–100)	83.3 (0.603)
Fever 48 h prior to survey (n = 208)									
Yes	42	0	2	2^a^	100 (92.3–100)	50.0 (6.8–93.2)	95.5 (84.9–98.7)	100 (15.8–100)	95.3 (0.646)
No	77	5	45	35	93.9 (86.3–98.0)	56.3 (44.7–67.3)	68.8 (59.3–77.2)	90.0 (78.2–96.7)	75.3 (0.504)
History of anti-malarial drug intake 1 week prior to the survey (n = 207)									
Yes	52	2	9	21	96.3 (87.5–99.0)	30 (14.7–49.4)	71.2 (60.0–80.3)	81.8 (48.2–97.7)	72.6 (0.306)
No	66	3	38	16	95.7 (88.0–98.5)	70.4 (57.2–80.9)	80.5 (70.6–87.6)	92.7 (80.6–97.5)	84.6 (0.678)

*CI* confidence interval, *PfHRP-2* *P. falciparum* histidine-rich protein-2, *pLDH* *Plasmodium* lactate dehydrogenase, *RDT* rapid diagnostic test, *PCR* polymerase chain reaction, *PPV* positive predictive value, *NPV* negative predictive value, *Kc* Cohen’s kappa coefficient;  % agreement was calculated by summation of the number of positives and negatives by both RDT and PCR divided by the total number of cases

* The agreement between RDT and PCR was significant for all categories with p < 0.001

^a^Parasite densities of these two cases were 80 parasites and 400 parasites/µL

### Sensitivity, specificity, positive, and negative predictive values of LM against nested PCR

Compared with nested PCR, LM had sensitivity of 37.6 % (95 % CI: 29.6–46.3), specificity of 97.6 % (95 % CI: 91.7–99.7), PPV of 37.6 % (95 % CI: 29.6–46.3), and NPV of 51.3 % (95 % CI: 43.2–59.2). The two types of tests showed a fair degree of agreement (61.7 %; *Kc* = 0.307, p < 0.001). A reduction in LM sensitivity was observed among children <10 years old, asymptomatic participants and those with history of anti-malarial drug intake. Although LM showed high sensitivity (93.5 %) for detecting symptomatic malaria, such sensitivity dropped to 8.5 % in case of asymptomatic malaria. On the other hand, LM maintained its high specificity for the detection of *P. falciparum* (Table 4).

**Table 4:** Sensitivity, specificity, PPV and NPV of LM for detecting *Plasmodium falciparum* against nested PCR as a reference method

	PCR + ve	PCR + ve	PCR−ve	PCR−ve	Sensitivity	Specificity	PPV	NPV	% Agreement*
	LM + ve	LM−ve	LM−ve	LM + ve	% (95 % CI)	% (95 % CI)	% (95 % CI)	% (95 % CI)	(*Kc*)
Overall	47	78	82	2^a^	37.6 (29.6–46.3)	97.6 (91.7–99.7)	95.9 (86.3–98.9)	51.3 (43.2–59.2)	61.7 (0.307)
Age (years) (n = 209)									
≥10	41	65	71	2	38.7 (30.0–48.2)	97.3 (90.5–99.7)	95.3 (84.5–98.7)	52.2 (43.5–60.8)	62.6 (0.329)
<10	6	13	11	0	31.6 (12.6–56.6)	100 (71.5–100)	100 (54.1–100)	45.8 (25.6–67.2)	56.7 (0.253)
Fever 48 h prior to study (n = 209)									
Yes	40	3	2	2	93.0 (81.4–97.6)	50.0 (6.8–93.2)	95.2 (84.2–98.7)	40.0 (5.3–85.3)	89.4 (0.386)
No	7	75	80	0	8.5 (3.5–16.8)	100 (95.5–100)	100 (59.0–100)	51.6 (43.5–59.7)	53.7 (0.084)
History of anti-malarial drug intake 1 week prior to the survey (n = 208)									
Yes	19	37	30	0	33.9 (22.9–47.0	100 (88.4–100)	100 (83.2–100)	44.8 (33.5–56.6)	57.0 (0.264)
No	28	40	52	2	41.2 (30.3–53.0)	96.3 (87.5–99)	93.3 (78.7–98.2)	56.5 (46.3–66.2)	75.6 (0.349)

*CI* confidence interval, *PCR* polymerase chain reaction, *LM* light microscopy, *PPV* positive predictive value, *NPV* negative predictive value, *Kc* Cohen’s kappa coefficient; % agreement was calculated by summation of the number of positives and negatives by both LM and PCR divided by the total number of cases

* The agreement between LM and PCR was significant in all categories with p < 0.05

^a^Parasite densities of these two cases were 80 parasites and 400 parasites/µL

## Discussion

Prompt malaria diagnosis is a key component of the national malaria control strategy in Yemen, which relies on the use of LM and RDTs. This study was designed to evaluate the diagnostic accuracy of these two methods against nested PCR in Mawza District, Taiz Governorate during the peak seasonal transmission. In the present study, the *Pf*HRP-2/pLDH RDT and LM showed a fair level of agreement in their performance to detect *P. falciparum* in the field, despite approaching 80 %. However, a substantial agreement was observed between RDT and LM for the detection of *P. falciparum* among febrile patients. This is consistent with a recent study [10] that reported a very good level of agreement between LM and CareStart™ HRP-2 RDT results among febrile patients. In the present study, RDT proved effective in detecting all LM-positive cases and in detecting a large proportion of LM-negative cases. The investigated RDT showed higher sensitivity than LM compared with nested PCR (96.0 vs 37.6 %), which is also higher than the sensitivity recommended by the WHO [5].

The good performance of the *Pf*HRP-2/pLDH RDT in the field is evidently shown by its ability to detect *P. falciparum* in all different degrees of microscopic parasite densities. Moreover, its sensitivity exceeds that of LM for parasite detection (93.9 vs 8.5 %) among afebrile participants, indicating its utility in active case detection. This could help in strategies for reducing malaria transmission by identifying asymptomatic carriers and their subsequent treatment. Similar findings of higher RDT sensitivity have been reported previously [21]. The WHO has recently demonstrated a good level of sensitivity of RDTs in low parasitaemia [5]. However, one should consider that not all RDT-positive cases correlated with those obtained by PCR. This in turn indicates that despite the better performance of RDT compared with LM, false positivity of RDT could not be ruled out. However, its performance is still superior to that of LM. In this respect, a moderate agreement (about 80 %) exists between RDT and PCR in detecting falciparum malaria among Yemeni patients in the field compared with a fair agreement (about 62 %) between LM and PCR. Most importantly, the *Pf*HRP-2/pLDH RDT showed a higher NPV than LM (90.4 vs 51.3 %) during the peak seasonal transmission of malaria. This is advantageous for the definite exclusion of malaria among patients, and the avoiding of unnecessary presumptive treatments. Given that the NPV is 100.0 % for the RDT and 40.0 % for LM among febrile patients, RDT-negative results for patients experiencing fever will be straightforward and will rationalize the prescription of anti-malarial drugs. Similarly, a very recent study [22] recommends the use of RDTs for diagnosis of suspected malaria among symptomatic pregnant women but not for asymptomatic cases in Papua New Guinea. A limitation of the present study is that it included only a subset of the negative samples for molecular analysis. However, the large difference between RDT-positive and LM-positive samples, which is still of suspected positivity and could be due to persistent antigenaemia (108 samples of the RDT-positive ones), helps to avoid or, at least, reduce any possible bias.

The superiority of RDTs compared with LM could be explained by the fact that sequestered *P. falciparum* missed by LM can be detected by RDTs because of the release of *Pf*HRP-2 by parasites and its circulation in the blood [23, 24]. Meanwhile, the low sensitivity of LM in the present study could also be attributed to the high proportion of asymptomatic, very low-parasite density malaria cases. RDTs targeting *Pf*HRP-2 have been suggested as a better alternative to LM in areas of low-density parasitaemia and their false positives compared with LM have been confirmed by PCR to be cases below the threshold detection of LM [25]. In addition to the diagnostic limitation imposed by microscopist expertise, the poor-quality LM in developing countries contributes to its low sensitivity in detecting low-parasite density infections. In Yemen, poor performance of LM for malaria diagnosis has been ascribed to low quality reagents, laboratory equipment and supplies [2]. LM of low quality has been reported to influence its sensitivity and specificity for malaria diagnosis [3].

In contrast, LM had higher overall specificity than RDT (97.6 vs 56 %) compared with the reference method. LM is still the gold standard for species identification and detecting the severity of malaria by quantifying parasitaemia and for differentiation of transmissible stages from those responsible for clinical disease [26]. Low specificity of the *Pf*HRP-2/pLDH RDT in the present study is in contrast to the high specificity (96.1 %) recorded for the CareStart™ HRP-2 RDT tested in an earlier study [10], which compared with microscopy for the detection of falciparum malaria among febrile patients. However, such low specificity is in agreement with a previous study comparing four brands of *Pf*HRP2-based RDTs for falciparum malaria diagnosis among febrile patients in Malawi, where specificity of 39–68 % was reported [27]. It is noteworthy that two cases were positive with both LM and *Pf*HRP-2/pLDH RDT but negative by the PCR reference method. Although these were considered as false-positive results compared to nested PCR as the reference method, unperceived factors contributing to the inhibition of PCR could not be ruled out. Moreover, PCR false-negativity has been documented in the literature compared to LM [12, 28–30]. The false positivity of the RDT in the present study could overestimate the prevalence rate by about 25 % as indicated by the PPV (76.9 %) compared with nested PCR. This is in agreement with the high false-positive rates of *P. falciparum* using *Pf*HRP-2-based RDTs reported from Congo [31, 32] and Burkina Faso [33]. The *Pf*HRP-2-based RDT false positivity and its relatively low PPV could be attributed to the persistence of *Pf*HRP-2 antigenaemia in the blood circulation for 4–5 weeks after parasite clearance with successful treatment [31, 33, 34]. The possible impact of persistent antigenaemia on the specificity of RDT investigated in the present could, in part, explain its dropped specificity to 30 % among patients with history of anti-malarial drug intake. Furthermore, malaria survey at the peak seasonal transmission, when prevalence rate is >10 % (Lina et al., unpublished data), may partially account for the low specificity of *Pf*HRP-2/pLDH RDT in the present study. Previous studies showed a negative correlation between the specificity of RDTs and malaria prevalence [31, 35–37]. False-positive results by *Pf*HRP-2/pLDH RDTs can lead to overdiagnosis and subsequent overtreatment, which may contribute to the emergence and spread of drug resistance [38]. Therefore, its combination with a more specific test is recommended. Furthermore, in addition to genus-specific pLDH, *P. falciparum*-specific LDH-based RDTs should be evaluated for screening of falciparum malaria in Yemen. This may help avoid the drawback of *Pf*HRP-2 RDTs resulting from persistent antigenaemia in blood after treatment and cure, minimizing the false positivity rate to reasonable and acceptable levels. However, *Pf*HRP2 positivity in the absence of *P. falciparum*-specific LDH or pan-specific LDH does not necessarily mean a false-positive result due to persistent antigenaemia [39]. Although PCR is the most sensitive and specific tool for malaria diagnosis [40], it is not practical for routine use in Yemen due to the limited resources.

Differences in sensitivity and specificity reflect on the estimation of falciparum malaria prevalence in the country, particularly among asymptomatic patients. In the present study, the overall prevalence of *P. falciparum* was three times higher when using *Pf*HRP-2/pLDH RDT compared with LM and 16 times higher among asymptomatic patients. In this context, Mappin et al. [41] reported a strong, non-linear relationship between malaria prevalence rates derived from the LM and RDTs. Higher RDT-based prevalence rates were also reported from Ethiopia and Tanzania, being two times and three times higher than those by LM, respectively [42, 43]. Although the sensitivity of RDTs may, to some extent, reflect the true prevalence of symptomatic as well as asymptomatic-treated cases that cannot be detected by LM or PCR in the field over a certain period, the prevalence estimates by RDT and LM need to be standardized if they are to be used for epidemiological purposes, such as mapping [41]. However, it poses a problem for case management, where unnecessary treatments could contribute to the emergence and spread of drug resistance. The better performance of RDTs over LM in field surveys has also been reported from the Brazilian Amazon [44] and Angola [45]. Overall, the findings of the present study suggest RDTs as a promising tool for epidemiological surveys in Yemen, even in low transmission settings and among asymptomatic carriers.

## Conclusions

The *Pf*HRP-2/pLDH RDT tested in the present study showed a better performance than LM in field survey for malaria, even in asymptomatic cases. It showed a moderate degree of agreement with nested PCR, with a high sensitivity. A major drawback of the RDT is that the high false-positivity rate limits its use as an independent diagnostic tool for malaria to avoid unnecessary overtreatment. However, its negative results totally exclude falciparum malaria among febrile patients as indicated by its 100 % NPV. This can have public health implications in educating healthcare providers and patients in endemic areas to perform RDTs in all cases of fever to exclude falciparum malaria before prescribing or taking anti-malarial drugs. The low sensitivity of LM indicates that a high proportion of malaria cases is missed, leading to an underestimation of the true malaria prevalence in the community. However, LM remains indispensable to species identification, differentiation of gametocytes from asexual stages and the assessment of the severity of the disease. Furthermore, it is still the gold standard for the diagnosis of symptomatic malaria. RDTs should be further investigated as rapid malaria-excluding diagnostics among febrile patients in endemic areas.
